# News and Notes

**Published:** 1912-05

**Authors:** 


### NEWS AND NOTES

### BEWARE.

Gustav Gebauer, formerly a reputable book seller, having obtained one of my order books, is collecting money from physicians in Georgia, North and South Carolina. None of these orders reach me and I am in no way responsible for them.

W. M. LEONARD, Publisher.

### AMERICAN MEDICAL EDITORS' ASSOCIATION.

The following papers will be presented on June 1st and 3rd at a meeting of the above Society at Marlborough-Blenheim Hotel, Atlantic City, N. J.

"The Advisability of Newspapers and Magazines Having Medical Editors on their Staffs."

By Edgar A. Vander Veer, M. D., Albany, N. Y.

"False Values in the Practice of Medicine."

By H. Edwin Lewis, New York.

"Science in Personal Journalism."

By T. D. Crothers, M. D., Hartford.

"Eugenics in the Medical Magazines."

By C. H. Hughes, M. D., St. Louis.

"The Province of the Editor in Medical Journalism."

By W. B. Snow, M. D., New York.

"Commercialism."

By C. F. Taylor, M. D., Philadelphia.

"Research Work in Life Insurance Medicine."

By invitation, Fred'l L. Hoffman, Newark, Statistician Prudential Ins. Co.

"Shifting Medical Conditions Confronting Medical Journalism."

By Invitation, E. A. Ayers, M. D., Prof. Emeritus Obstetrics, New York Polyclinic.

"Medical Journalism from a Disinterested Standpoint."

By Albert E. Sterns, M. D., Indianapolis.

"What Doctors Read and Write."

By Erwin Reissman, M. D., New York.

"Independent Medical Journalism and the Task of the Independend Editor of To-day."

By W. J. Robinson, M. D., New York.

"Differential Diagnosis Between the "Write-up" and an Honest Article on a New Remedy."

By Invitation, H. S. Baketel, M. D., New York.

"The Medical Editor of a Daily Newspaper, His Duties and His Educational Opportunities."

By A. S. Burdick, M. D., Chicago.

"Medical Expert Testimoney."

By R. B. H. Gradwohl, M. D., St. Louis.

"Book Reviews."

By Arnold Snow, M. D., New York.

"State Board Examination Questions and Answers in Medical Journals."

By Hills Cole, M. D., New York.

"Subscription Getting."

By G. Strobach, M. D., Cincinnati.

"Laboratory Experiments versus Clinical Experience as a Method in Determining the Therapeutic Value of a Remedy."

By John W. Wainwright, M. D., New York.

"Anonymous vs. Person Journalism.

By Chas. A. Wingerter, M. D., Wheeling, W. Va.

"Subject to be Announced."

By W. A. Young, M. D., Toronto, Canada.

"How the Medical Press Can Co-operate with the Manufacturers for the Proper Introduction of New Materia Medica Science and Brands of the same to Commerce."

By Invitation, F. E. Stewart, Ph. G., M. D., Prof. of Materia Medica, School of Pharmacy, Medico-Chirurgical College, Philadelphia.

---

### ARMY MEDICAL CORPS EXAMINATIONS.

The Surgeon General of the Army announces that preliminary examinations for the appointment of first lieutenants in the

Army Medical Corps will be held on July 15, 1912, and September 3, 1912, at points to be hereafter designated.

Full information concerning these examinations can be procured upon application to the "Surgeon General, U. S. Army, Washington, D. C." The essential requirements to securing an invitation are that the applicant shall be a citizen of the United States, shall be between 22 and 30 years of age, a graduate of a medical school legally authorized to confer the degree of doctor of medicine, shall be of good moral character and habits, and shall have had at least one year's hospital training, after graduation. The examinations will be held concurrently throughout the country at points where boards can be convened. Due consideration will be given to localities from which applications are received, in order to lessen the traveling expenses of applicants as much as possible.

The examination in subjects of general education (mathematics, geography, history, general literature, and Latin) may be omitted in the case of applicants holding diplomas from reputable literary or scientific colleges, normal schools or high schools, or graduates of medical schools which require an entrance examination satisfactory to the faculty of the Army Medical School.

In order to perfect all necessary arrangements for the examination, applications must be complete and in possession of the Adjutant General at least three weeks before the date of examination. Early attention is therefore enjoined upon all intending applicants. There are at present sixty-eight vacancies in the Medical Corps of the Army.

---

### MEDICAL INTERNE. GOVERNMENT HOSPITAL FOR THE INSANE, JUNE 5TH, 1912.

The United States Civil Service Commission announces an examination on June 5, 1912, at the places mentioned in the list printed hereon, to secure eligibles from which to make certification to fill two or more vacancies in the position of medical interne, Government Hospital for the Insane, Washington, D. C., at $600 per annum, with maintenance, and vacancies requiring similar qualifications as they may occur in that hospital, unless it be found to be in the interest of the service to fill such vacancies by reinstatement, transfer, or promotion.

The positions are tenable for one year, and pay $50 a month and maintenance. At the end of six months, however, during which time a postgraduate course in mental and neurological diagnostic methods, etc., is given, an examination is held, and promotions to the next grade, assistant physician, at $75 a month and maintenance, are made. Beyond this there is regular advancement for men whose services are satisfactory. The Government Hospital for the insane has over 2,900 patients and about 750 employees to care for. In addition to the general medical practice offered, the scientific opportunities are excellent and the clinical opportunities in neurology and psychiatry are unsurpassed.

As considerable difficulty has been experienced in filling vacancies in the position of medical interne in the Hospital Service during the past several years owing to the limited number of eligibles available, qualified persons are urged to enter this examination.

The examination will consist of the subjects mentioned below, weighted as indicated:

| Subjects. | Weights. |
| --- | --- |
| 1. Letter writing (the subject matter on a topic relative to the practice of medicine) ---------------------------- | 5 |
| 2. Anatomy and physiology (general questions on anatomy and physiology, and histologic or minute anatomy ------ | 10 |
| 3. Chemistry, material medica, and therapeutics (elementary questions in inorganic and organic chemistry; the physiologic action and therapeutic uses and doses of drugs) ---------------------------------------- | 15 |
| 4. Surgery and surgical pathology (general surgery, surgical diagnosis, the pathology of surgical diseases) ---- | 20 |
| 5. General pathology and practice (the symptomatology, etiology, diagnosis, pathology, and treatment of diseases - | 25 |
| 6. Bacter ology and hygiene (bacteriological methods, especially those relating to diagnosis; the application of hygienic methods to prophylaxis and treatment) ---- | 10 |

7. Obsterics and gynecology (the general practice of obstetrics, diseases of women, their pathology, diagnosis, symptoms and treatment, medical and surgical) ------ 15

Total ---------------------------------------- 100

Applications will be accepted only from persons who indicate in answer to question 18 of the application form that they have been graduated from reputable medical college.

Applicants must not have been graduated more than two years prior to the date of examination unless they have been continuously engaged in hospital, laboratory or research work along the lines of neurology or psychiatry since graduation, which fact must be specifically shown in the application.

Both men and women will be admitted to the examination, although there are no vacancies for women at present. Applicants must be unmarried.

Age limit, 20 years or over on the date of the examination.

This examination is open to all citizens of the United Stated who comply with the requirements.

This announcement contains all information which is communicated to applicants regarding the scope of the examination, the vacancy or vacancies to be filled, and the qualifications required.

Applicants should at once apply either to the United States Civil Service Commission, Washington, D. C., or to the secretary of the board of examiners at any place mentioned in the list printed hereon, for application and examination Form 1312. No application will be accepted unless properly executed and filed with the Commission at Washington. In applying for this examination the exact title as given at the head of this announcement should be used in the application.

As examination papers are shipped direct from the Commission to the places of examination, it is necessary that application be received in ample time to arrange for the examination desired at the place indicated by the applicant. The Commission will therefore arrange to examine any applicant whose application is received in time to permit the shipment of the necessary papers. Issued April 20, 1912.

### EXCERPTS FROM

### CLINICAL SOCIETY OF THE NEW YORK POLYCLINIC MEDICAL SCHOOL AND HOSPITAL. ING OF APRIL 1ST, 1912.

Three Cases of Sterility Secondary to Adnexa Disease: Cured by Operation.—Presented by Dr. Henry V. Holcomb.

These three cases were of interest as showing what could be accomplished by conservative work.

The first case was a woman 19 years of age who had had a previous miscarriage in the sixth week of pregnancy and gave symptoms showing pelvic trouble. Operation by Dr. Child showed both ovaries bound down by adhesions, a cyst attached to the left tube, and the tubes closed. The cyst was evacuated, the adhesions broken down, and the tubes probed with a fine bougie their entire length. Convalescence was uneventful. Four months after the operation she was free from any abdominal symptoms. Menstruation regular. She later showed symptoms of a floating right kidney which was anchored by three silk stitches. Convalescence from this operation was also uneventful. February 2nd she had her last menstruation and by the end of March she began to have morning vomiting with swelled breasts, and all the evidences of pregnancy. She had a precipitate delivery of a seven and a half months foetus, in the following August. The child died, but the mother is alive and well.

The second case was somewhat similar: the patient came with all the abdominal symptoms of inflammation of the appendages. Dr. Child found on operation that both ovaries were prolapsed and bound down by adhesions, occluding the tubes. After her operation she returned home well. One month after the operation she became pregnant and was delivered of a full-time child.

The third case was a woman of 31 years of age who had been sterile for thirteen years. She had a former child and was anxious to have another. She had pelvic pains bilaterally, which had been present for the previous four years. Examination showed a retroverted uterus, and tender appendages. Local treatment gave no relief. Operation in 1909 by dilation, curettage, followed by laparotomy showed the same adhesions of ovaries, with closed fimbriated extremities as in the other two cases. The same method of treatment was followed, and the patient made an uneventful recovery. She gained in weight after the operation, and in the following November was delivered of a normal child.

None of the above three cases came to be operated upon for sterility, but Dr. Holcomb laid stress upon the desirable outcome of cases who have primarily adnexal trouble, and in the correction of these secured the desired pregnancy. Dr. Holcomb said that as far as could be ascertained these cases were free from gonorrhoea.

Dr. Child stated that the cases reported were very interesting to those who were striving for the correction of sterility where the fertility of the husband was unquestioned. He had felt that it was important to thoroughly probe the tubes in these cases to insure an absolute patency. With the adhesions of the adnexa corrected, malpositions replaced, and the toilet of the uterus complete, he thought that there was a field of work open to the careful and discriminating surgeon, which would be effective in overcoming some hopeless cases of sterility.

Dr. Holcomb, in closing the discussion, said that the tubes were absolutely closed and the fimbria clubbed so that in the ordinary course of events pregnancy would have been impossible.

Specimen of an atrophied Right Kidney with Greatly Enlarged Opposing Kidney Non-Functionating.—Presented by Dr. Chetwood.

Dr. Chetwood illustrated the value of the colorimeter test in measuring the capacity of the kidney function. His patient on cystocopic examination showed apparently normal ureteral orifices from both kidneys. The left ureter was easily catheterized, withdrawing urine of low specific gravity, deficient in urea and a number of leucocytes: pus though present was in insignificant amount. The right ureter was rigid and impossible to catheterize and the cystoscope showed a certain amount of purulent granular material slowly exuding. The patient was then subjected to the colorimeter test, made by injecting I c. c. of phenol-sulphone-phthalein, after having previously emptied the bladder. At one and two hour intervals the urine was collected from the bladder. The returned percentage of color was so low that the individual kidneys were tested separately, resulting in an absence of color on the right side and a very low color return on the left

Operative treatment seemed necessary, and after taking X-ray pictures to rule out the possibility of a calculus, the patient was operated upon. Operation showed a vestiginary right kidney with a ureter entering the bladder but having a sacculated distal end. On the left side, which was unsuspected, there was a perfectly normal ureter below, while above was a large ureteral sac almost as large as the small intestine, which entered the kidney. A large peri-nephritic pus sac occupied one side of the kidney, and practically no kidney tissue remained even in this organ to secrete. The patient could not possibly have lived as proved by the colorimeted test and subsequently verified by autopsy.

Dr. Sinclair said that in Dr. Chetwood's case there had been a slight previous injury many years ago, but aside from blood in the urine for a few days, no recurrent symptoms ot any kind had occurred.

Dr. Weyth said a very interesting phase of the case was the extremely limited excretory area eliminating urea. Considering the good health the patient enjoyed he thought the skin must have employed an important part in the excretion of urea.

---

Of so great interest to Georgians is this special Bulletin, that we desire to reproduce in full the account of the unveiling of the memorial to Crawford W. Long:

### SPECIAL BULLETIN, UNIVERSITY OF PENNSYLVANIA, PHILADELPHIA, PA., U. S. A.
### MEMORIAL TO DR. CRAWFORD W. LONG, OF THE CLASS OF 1839, MEDICAL.

An Account of the Ceremonies of the Unveiling of a Bronze Medallion in the Medical Building on March 30, 1912, to the Memory of Crawford W. Long, Who First Used Ether as an Anaesthetic in Surgery on March 30, 1842.

In the summer of 1910, at the British Medical Association meeting in London, Mrs. Frances Long Taylor presented original documents proving that her father, Crawford W. Long, gave ether as an anaesthetic for surgical purposes in 1842, four years before any other claimant for the discovery. While this had been known by authorities on anaesthesia, she felt that her father's memory should have wider recognition, and it has been her self-appointed and filial task to place the facts before the profession lest her father's modesty and self-effacement should result in their being neglected or forgotten.

This made his fame secure in England, and when the University of Pennsylvania decided in the same year to celebrate the seventieth anniversary of this great medical discovery made by her graduate, by unveiling a memorial bronze to his memory, Mrs. Taylor felt that her life's ambition had at last been gratified and that the whole world would now recognize his undisputed claim.

The form of the memorial is a medallion, which enabled the artist to put on record the story of the first operation in plastic form and also to use as a decoration an appropriate inscription, neither of which would be possible in a bust.

The only available pictures were a crayon drawing of Dr. Long at twenty-six and a steel engraving showing a man of sixty. They have little in common so far as likeness is concerned, but as he was a graduate of only two years' standing when his first operation was performed, the crayon drawing, crude as it was, formed the basis on which the head was modelled. Work was started in the autumn of 1910 and has been continued throughout both winters, passing through many vicissitudes and changes of arrangement and composition.

The completed medallion shows the young doctor bending forward over a recumbent patient, dropping ether from a bottle held in the right hand on the towel that partly covers the patient's face and watching intently the patient's respiration. A spray of poppy leaves and pods rise from either side of the plate, bearing the words, "Class of '39 Pennsylvania," while the circular inscription above his head runs in two lines, "To Crawford W. Long, First to Use Ether as an Anaesthetic in Surgery, March 30,

1842," "From His Alma Mater." In the field to the left is the date of his birth and death, 1815 and 1878.

R. TAIT MCKENZIE, M. D.

### MEMORIAL TO DR. CRAWFORD W. LONG.

### THE CEREMONIES AT THE UNVEILING OF THE TABLET.

Dr. Crawford Williamson Long, who first made use of ether as an anaesthetic for surgical purposes, on March 30, 1842, was memorialized on Saturday afternoon, March 30, 1912, when a handsome gilt bronze medallion was unveiled in his honor. The exercises were held in the Medical Building of the University of Pennsylvania. Addresses were made by Dr. J. William White, of the University, and Dr. J. Chalmers Da Costa, of Jefferson Medical College. The medallion was modeled by Dr. R. Tait McKenzie, of the University, and represents Dr. Long as a young man administering ether for the first time to a patient about to be operated upon.

Provost Edgar F. Smith presided and introduced the speakers, after a brief invocation of Deity offered by Rev. Robert Johnston, of the Church of the Saviour, of this city. The tablet was unveiled by Mrs. Florence L. Bartow, a daughter of Dr. Long, after the address of Dr. J. William White. Dr. J. Chalmers Da Costa followed and the ceremonies closed with a brief reply by Hon. Samuel J. Tribble, who thanked the University on behalf of the family and the State of Georgia, for the honor the University had conferred upon its illustrious graduate. The presence of three distinguished Southern ladies, Mrs. Frances Long Taylor, Mrs. Alexander O. Harper and Mrs. Florence L. Bartow, the daughters of Dr. Long, added great interest and dignity to the occasion. They came from Athens, Georgia, for the express purpose of attending the ceremonies, and during their stay in Philadelphia were the guests of the University.

The addresses follow:

### THE RELATION OF THE UNIVERSITY OF PENNSYLVANIA TO THE EMPLOYMENT OF ANAESTHESIA IN SURGERY.

By J. William White, M. D. LL. D.

We have come here to-day to do honor to the memory of a son of Pennsylvania who was the pioneer—who actually led the world—in what was, perhaps, the most momentous attack upon pain and suffering—and, indirectly, upon disease itself—ever made in the history of mankind. That specific subject I shall not undertake to deal with in detail. But, as a preface to it, I should like to call attention to the way in which the labor of others, and especially of other sons of Pennsylvania, helped first to prepare the field for the introduction of anaesthesia, and later to profit by it and by the discoveries that followed and largely resulted from it. The interdependence of the sciences has long been recognized, as has the often surprising way in which what is called "pure" science has led to practical results as beneficient as they were unexpected. It seems a far cry from the experiments of our founder, Benjamin Franklin, with his celebrated kite, to the time, nearly one hundred years later, when mankind

"'Mid deepening stillness watched one eager brain,
With Godlike will, decree the Death of Pain."

And yet these two occurrences, of such tremendous importance to humanity, are connected—and not remotely—by a chain of scientific events; and some of the most important links of that chain were forged by our teacher and alumni.

If we study the history of the great scientific achievements of the past we shall be struck with the fact that their true origin is almost always to be found in efforts usually distant in time and place, and often made in some other field of human endeavor.

The only addition of the nineteenth century to medical science that can be compared with anaesthesia as a boon to humanity is the recognition of asepsis as a surgical principle. It is easy to show that Lister's grand conception of the relation of bacteria of the diseases of wounds can be traced directly to the attempts of a provincial French chemist (Pasteur by name) to determine why a certain fungus affected the two varieties of tartaric acid differently. In fact, to that piece of chemical work and the further researches to which it gave rise, may also be traced not only the magnificent development of aseptic surgery, but likewise our present positive knowledge of the microbic character of infections and our modern treatment of those diseases by vaccines, serums and antitoxins.

Examples might be multiplied. The X-rays, now absolutely essential to both surgeon and physician, were found by Roentgen while investigating the effect of electricity upon gases, with not the slightest idea of discovering anything to use in the diagnosis or treatment of disease. Bacteriology, itself the foundation of medicine and surgery, is largely based on the action of bacteria to certain aniline dyes which were originally isolated merely to establish their chemical properties and commercial uses. The compound microscope, without which medicine would be where it was in the middle ages, came from experiments by physicists on the refraction of light, with no adequate thought of the new world it has revealed to us. Some entomologists who, for purposes of classification, were studying the wings and the skin covering and appendages of mosquitos, accumulated facts, that applied by others, brought the death rate from yellow fever in Havana from 36,000 in a single epidemic (1878) to zero in the years 1902, '03, and '04. M. and Mme. Curie, in searching for radium, had no thought that it, like the X-rays, might cure superficial cancers. Metchnikoff, a biologist, when he studied the swallowing and destruction of microbes by the living cells of a small crab, did not foresee the manifold applications of the principle of phagocytosis to the cure of disease, and never dreamed of the opsonin treatment and tests of to-day. When (in 1846) Joseph Leidy, afterwards for many years our Professor of Anatomy, and until his death the greatest American naturalist, found in a slice of boiled ham, from which he had partly made his dinner, the little immature worm, the trichina spiralis, he did not for a moment suspect the significance of the discovery, or that it would ultimately involve hundreds of millions of money and even the peace of nations.

And so, it might be illustrated, almost indefinitely, that the piling up of facts by those born with the impulse to delve into the unknown, or by those with the instinct to bring order out of confusion, and to group, arrange and classify physical truths, and living beings, has always gone on, as it is going on now, and that the great "discoveries" that constitute the milestones of medical and scientific progress, are always the result of the gradual accumulation of material usually made with no reference to its beneficient employment.

But there is something else than "facts" needed before the discovery comes. Ether may stand—as it did stand—for three hundred years on the chemists' shelves while the tortures and agonies of injury, of disease, even of physiological processes like parturition, go on unalleviated. Men there may be during those centuries with minds as keen and abilities as great as those of any who follow them, but the two—the physical substance and the minds cognizant of its existence—remain as relatively alien and unproductive as flint and steel in the absence of contact, or as the two poles of a battery without a uniting medium. That medium is difficult of precise definition. For want of a better term, it may, for my present purpose, be spoken of as a favoring intellectual—in this case a scientific—atmosphere. This is always a slow formation. The facts are there; the men are there. But the currents of thought, the connecting medium, are absent. By the time the "atmosphere" has formed, when the world is as it were ready for the discovrey, there have been almost invariably (perhaps under unconscious telepathic influences of which we as yet know nothing), several minds turning or groping in the same direction.

One single example must suffice. The crowning intellectual achievement of the nineteenth century was the enunciation—with convincing proofs—of the doctrine of evolution by Charles Darwin. The way had been blazed for him by Goethe and Erasmus Darwin and Lamarck, but their somewhat vague ideas had been allowed to fade into forgetfulness. When, however (in 1858), after spending fourteen years in collecting evidence, he was almost ready for publication, he received from a friend, Alfred Wallace, living at the antipodes, who had no knowledge of Darwin's work, a manuscript that contained observations, reasonings and conclusions, that precisely paralleled his own. In November, 1859, he gave his immortal work on "The Origin of Species" to the world. On October 11th, of the same year, a month previous, Prof. Joseph Leidy, in his introductory lectures to the Medical Class of this University, said: "We are accumulating facts from which our successors may, perhaps, derive positive opinions in relation to the earliest history of organized beings, whether their species or various forms had a unique or prural origin, and whether or not the race of one age is the descendant of that which preceded it."

As we look back, with these ideas in mind, we find that our predecessors at Pennsylvania certainly had their share in the accumulation of the facts and in the formation of the atmosphere that jointly led to the use of ether in surgery.

In justifying this statement, I must begin (as we always begin here) with Benjamin Franklin, the greatest American of all time. That he founded the University (1740), advanced in every way the cause of general education, built and started the first American General Hospital (1775), and did all the work as a statesman, a scientist, and an experimentalist with which the world is now so familiar, may not seem to have much direct relation to our subject, except that it was all undoubtedly effective in fostering every sort of intellectual advancement. His most valuable work, however, in preparing the field for the introduction of anaesthesia, was in the vigorous and successful fight he waged against the bigotry, fanaticism and ignorance which then, as now, were opposed to inoculation for smallpox, the greatest scourge of that and of many preceding centuries. Although inoculation had been introduced into this country in 1721, it was still bitterly inveighed against, partly, to our shame be it said, by the reactionaries, the "stand-patters," in our own profession, and largely by the clergy, who preached against it—as they did a century and more afterward against the abolition of pain—as "subverting the decrees of Providence and resisting the punishments of God." In 1732 a Reverend Mr. Massey preached from the text of Job 2: 7: "So went Satan forth from the presence of the Lord, and smote Job with sore boils from the sole of his foot unto his crown," concluding that "the cutaneous disease of Job was produced by inoculation from the hands of the devil, and the whole are of infernal invention."

One hundred and more years later a clergyman wrote to Sir James Simpson, who was employing anaesthesia in childbirth, and characterized it as "a decoy of Satan, apparently offering itself to bless women, but in the end destined to harden society and rob God of the deep earnest cries which arise in time of trouble, for help."

In breaking down by precept, example and widepsread proclamation of his views this biased and besotted antagonism to a great public benefaction, Franklin was as surely aiding in the discovery of anaesthesia and its use as if he had foreseen it.

The "most notable American medical essay of the eighteenth century," according to an unprejudiced Bostonian, was the Discourse at the Commencement of this University in 1765, given by Dr. John Morgan, which led to the immediate establishment of this Medical School, the first in America. The same historian calls Morgan the "grandfather," as Benjamin Rush was the "father" of American medicine.

No single agency contributed more to the preparation for anaesthesia than Morgan's teachings as to medical education with its fundamental relation to the natural sciences, chemistry, anatomy and physiology. It was begun on that basis in this country and in this school in the same year (1765) and has been carried on through all the intervening years on the same broad principles, through the period of Dr. Long's attendance here and down to the present day. Its influence has been nation-wide—often world-wide—and as a factor in bringing about the condition of medical science to-day, it can never be ignored.

In 1768 Benjamin Rush was elected a professor in this University. He was not only, as Mumford has called him, the "Father of Medicine," in this country. He was the father of Experimental Medicine, the founder of Scientific Medicine, almost the first distinguished practitioner to evince, in the midst of the fog which then enshrouded our profession, the desire to discover the underlying principles of disease and treatment. He was the most brilliant teacher of the day, and it can be readily understood that the man who, during the great epidemic of yellow fever, in 1793, when thousands were fleeing from this stricken city, could write to a friend: "I have resolved to stick to my principles, my practice, and my patients to the last extremity," was not without influence on those who surrounded and on those who followed him. He wrote, in 1812, that he had reached the conclusion that "pain does not accompany child-bearing by an immutable decree of Heaven," and that he hoped "that a medicine would be discovered that should suspend sensibility and leave irritability or the power of motion unimpaired, and thereby destroy labor-pains altogether." It may well be that the echoes of such teaching reached and directly inspired Crawford Long, who began the study of medicine only twenty-five years after the expression of this prophetic hope. Contemporaneous with or following Rush came Woodhouse (1792), who helped to break down the old phlogistic theory which stood in the way of all chemical advance, and who demonstrated that oxygen was given off by living plants —a fact of immense importance in the later studies of animal heat and respiration, and, therefore, in all our present views of the processes of both health and disease. We cannot claim Joseph Priestly, the discoverer of oxygen, as a "son of Pennsylvania," but as he was offered the chair of Chemistry in 1794, and declined only on account of advancing years, and as he was in frequent correspondence with the professors and alumni of that period, he may, perhaps, with propriety, be associated with the men I have described. Robert Hare (1818) added the Voltaic pile and the oxyhydrogen blowpipe to the equipment, first of the physicists and then of the manufacturers of the world. Young, in his graduation thesis, in 1803, determined the presence of a digestive acid and the ferment action of the gastric juice thirty years before the classical experiments of Beaumont upon St. Martin. John Redman Coxe (1809), though a medical man, was the first of all Americans after Franklin, to propose a plan for electric telegraphic communication; while Philip Syng Physick (1805) was the first surgeon in the world to use absorbable animal ligatures. George B. Wood (1835) built a still-existing monument to his memory by putting forth the Dispensatory of the United States; Chapman (1816), Gibson (1819), Barton and surgeon. Incomplete work is another great handicap to suc-
stomach. Uriseption in the proctoclysis in gall bladder cases
(1789) and others, made the school still more famous by their work and their teachings. In 1838, when Dr. Long came to us, the reputation of the School had so extended, that our Alumni— the pupils of the men I have named—were filling the most important chairs in the chief medical colleges of the country, two at Harvard, two in New York, two in Winchester, Virginia; two in Lexington Kentucky; four in Baltimore, six in Charleston, and, as has always been the case, some of our very best with our younger sister, the Jefferson Medical College. There were then eighteen medical journals in America, and ten of them were originated and edited by our graduates. With almost no exception the systematic treatises then in use in Medicine, Surgery, Obstetrics, Materia Medica and Therapeutics, had been written by our professors. In 1838 there had been put forth from this School textbooks on Anatomy, 11 (the last, published that very year, having been "Practical Lessons in Anatomy," by Dr. Hayes Agnew); on Surgery, 11; on Medicine, 15; on Midwifery, 12; on Materia Medica and Therapeutics, 18, including the Dispensatory. In addition may be named one of the first American Medical Dictionaries, one of the first compendiums, and 31 important American editions of European authors with notes and comments intended to make them more useful to American students.

It may certainly fairly be said that when Crawford Long came here at the age of 23, he found, as he could have found nowhere else in America, the scientific traditions, the intellectual stimulus to original thoughts and deeds, the "atmosphere," in other words, that was favorable, probably essential to his later achievement.

It is tempting to continue and try to show by our records that, while Crawford Long's name, and, therefore, the name of this School, are identified with the greatest contribution to Medical Science yet made by America, there has been done here durin the seventy years that have elapsed since that memorable March 30th of 1842, much work that has already notably increased the sum of useful knowledge, and much more that will doubtless prove to be the foundation of some now unforeseen and unimagined addition to Medical Science.

Gerhard, who first clearly differentiated typhus and typhoid fevers; Pepper, with his fundamental examination of the pathology of pernicious anemia; H. C. Wood, who first led the profession of the country to the intelligent study of the physiological action of drugs; Wormley, with his classical work on the microchemistry of poisons; Mills, with his researches into cerebral localization; Flexner, with his fruitful investigation into the cause of bacillary dysentery; Osler, with his study of the hematozoon of malarial fever; Guiteras, with his description of filariasis; Allen Smith, with his discovery of the hookworm disease; Leo Loeb, with his experiments in tumor-transplantation,—these are but a few—not one in twenty—of the names and achievements that jostle one another for recognition, some of them belonging to the generation just reaching scientific maturity. The list would, however, be inexcusably incomplete without mention of that great contribution to general science made by Reichert and Brown, and recently published by the Carnegie Institution—a work which extends the doctrine of evolution to the physical construction of the protoplasmic molecules of animals and plants, and opens an endless field of application to the difficult problems of specific growth, cellular and six differentiation and to the explanation of metabolism, immunity, tumor growth and the most intricate phases of physiology and pathology. The great teachers, the distinguished practitioners, the writers of textbrooks that have been the guides and consultants of thousands of medical men exend in an unbroken line from Rush, Wistar, Horner, Barton, Chapman, through Stillé, Carson, Agnew, Pepper, Leidy, Penrose, Wood, Goodell, to the present day.

It is a gratification to think that we are participating in exercises destined to add beyond cavail or future question the name of Crawford Long to that list of distinguished Pennsylvanians, who have well and faithfully served their profession and their country. There it rightfully belongs, and we may feel that his never-to-be-forgotten act will be more than ever an example and a source of pride to successive generations of our students and alumni. So great a feat may never be duplicated. It is not given to many to take the first step in wiping out immeasurable agony and suffering. Any yet—who knows? Lord Lister told me that in his very earliest days in Ediburgh, when he was still uncertain whether to remain there or begin his work elsewhere, he consulted Mr. Syme, who was then the leading surgeon of Great Britain. The latter told him that he would probably do well to stay there, but remarked that it really seemed as though there were not much left to do in the way of advancing surgical science, little thinking at the time that the young man he was talking to, his future son-in-law, would almost alone and unaided, effect the greatest revolution in surgery, and bring about the greatest step in advance, which has been made since Harvey discovered the circulation of the blood.

It would be presumptuous folly to assert that we are as yet beyond the threshold of our science. With each addition to human knowledge comes the possibility of some new, perhaps some overwhelming revelation of usefulness to humanity. The keenest foresight, the most daring imagination cannot penetrate the first and nearest of the endless vistas that stretch before us. Some day anaesthesia and asepsis—immeasurably the greatest advances of this age—may have only historic interest. But it is well to thing that in looking back, the names of Crawford Long and of the University of Pennsylvania will always be associated with the first of these and that, if we look forward, there is every reason to hope and believe that those names will be an inspiration to the thinkers, the investigators, and the discoverers of the future.

